# Application of polydopamine-modified triphasic PLA/PCL-PLGA/Mg(OH)_2_-velvet antler polypeptides scaffold loaded with fibrocartilage stem cells for the repair of osteochondral defects

**DOI:** 10.3389/fbioe.2024.1460623

**Published:** 2024-09-19

**Authors:** Renyi Cheng, Tao Xie, Wen Ma, Peishen Deng, Chaofeng Liu, Yuchen Hong, Changyu Liu, Jinjun Tian, Yanhua Xu

**Affiliations:** ^1^ Department of Orthodontics, Affiliated Stomatology Hospital of Kunming Medical University, Kunming, Yunnan, China; ^2^ Yunnan Key Laboratory of Stomatology, Kunming, China; ^3^ Department of Oral and Maxillofacial Surgery, Affiliated Stomatology Hospital of Kunming Medical University, Kunming, Yunnan, China; ^4^ Second Clinic, Affiliated Stomatology Hospital of Kunming Medical University, Kunming, Yunnan, China

**Keywords:** fibrocartilage stem cells, velvet antler polypeptide, tissue engineering, triphasic scaffold, osteochondral defects

## Abstract

Articular cartilage defects often involve damage to both the cartilage and subchondral bone, requiring a scaffold that can meet the unique needs of each tissue type and establish an effective barrier between the bone and cartilage. In this study, we used 3D printing technology to fabricate a tri-phasic scaffold composed of PLA/PCL-PLGA/Mg(OH)₂, which includes a cartilage layer, an osteochondral interface, and a bone layer. The scaffold was filled with Velvet antler polypeptides (VAP), and its characterization was assessed using compression testing, XRD, FTIR, SEM, fluorescence microscopy, and EDS. *In vitro* investigation demonstrated that the scaffold not only supported osteogenesis but also promoted chondrogenic differentiation of fibrocartilage stem cells (FCSCs). n vivo experiments showed that the tri-phasic PLA/PCL-PLGA/Mg(OH)_2_-VAP scaffold together with FCSC, when transplanted to animal models, increased the recovery of osteochondral defects. Those results demonstrate the promising future of illustrated tri-phasic PLA/PCL-PLGA/Mg(OH)_2_-VAP scaffold loaded with FCSCs as a new bone and cartilage tissue engineering approach for osteochondral defects treatment.

## 1 Introduction

The osteochondral defects are highlighted by the occurrence of joint cartilage as well as subchondral bone damage at the same time (Jacob et al., 2020); while they are typically caused by osteoarthritis, injury, or other factors. Since there are no blood vessels and lymphatic vessels in the joint environment, they limit the nutrients’ supply and the access of progenitor cells needed for the spontaneous healing of osteochondral defects (Huey et al., 2012). Current conventional clinical treatments are limited to microfracture, autologous chondrocyte implantation (ACI), as well as osteochondral autografts and allografts (Mithoefer et al., 2009; Peterson et al., 2010; Raikin, 2009). Nevertheless, these approaches may lack efficiency in the resultant formation of fibrocartilage, which may have not yet been fully characterized from the bioactivity perspective; in acquiring chondrocytes and eventually in their potential rejections (Galperin et al., 2013; Tuan, 2007; Bal et al., 2010). To overcome those challenges, osteochondral tissue engineering is an area in the current research field. Through tissue engineering methods, it is possible to explore the development of improved osteochondral tissue regeneration procedures, which may be the source of inspiration for modern new clinical strategies around the world (Chen et al., 2023).

The articular tissue is composed of hyaline cartilage layer, calcified cartilage layer and subchondral bone layer with layered structure (Fu et al., 2021). Articular osteochondral defects cause damage to both articular cartilage and subchondral bone at the same time. Therefore, it is necessary to select specific repair strategies for articular osteochondral (Nooeaid et al., 2012). Biomimetic scaffolds are a direction in the research of osteochondral tissue engineering systems. It is necessary to design scaffolds that can replicate the layered structure of articular cartilage tissue, where the dual interface of the cartilage layer and the subchondral bone layer will meet the repair needs of the cartilage surface and subchondral bone (Mohammadinejad et al., 2020). The calcified cartilage layer is located between mature cartilage and bone, serving as a transitional layer within the osteochondral tissue. This layer acts as a barrier to maintain the respective growth microenvironments of bone and cartilage. (Wei and Dai, 2021). Therefore, scaffolds for osteochondral tissue engineering have an osteochondral separation layer to mimic the role of the calcified cartilage layer.

The pore size of scaffolds significantly influences the biological characteristics of seeded cells. Scaffolds with small pore sizes (150–300 μm) can induce cell aggregation, promote chondrogenic differentiation of seeded cells, and enhance cartilage matrix synthesis (Nava et al., 2016; Nehrer et al., 1997; Stenhamre et al., 2011). The optimal pore size range for bone regeneration is 300–500 μm. Pore sizes smaller than 300 μm may lead to hypoxia, affecting nutrient transport and bone formation (Karageorgiou and Kaplan, 2005; Kuboki et al., 2001; Yang et al., 2023). Studies have shown that a bilayered PLGA scaffold with pore sizes of 100–200 μm in the cartilage layer and 300–450 μm in the bone layer can achieve optimal osteochondral defect repair (Duan et al., 2014).

The scaffold materials for osteochondral tissue engineering can be roughly divided into four categories: metals, ceramics, polymers, and hydrogels (Ramzan et al., 2023). Polymers, such as polylactic acid (PLA) and polycaprolactone (PCL), are widely used due to their excellent biocompatibility and ease of modification. To overcome the inherent drawbacks of single-type materials, researchers can develop polymer composites (Ostafinska et al., 2017). In this approach, another commonly used material is poly(lactic-co-glycolic acid) (PLGA), which is preferred for its high biocompatibility, adjustable degradation rate, and FDA approval (Hu et al., 2018). However, the main drawback of PLGA is the production of lactic acid, which can lead to local acidic pH, causing inflammation. The incorporation of magnesium hydroxide (Mg(OH)2) into composites successfully neutralizes lactic acid, thereby regulating pH fluctuations while promoting cartilage regeneration through the key element magnesium (Park et al., 2018; Yao et al., 2021b; Li et al., 2024).

Velvet antler polypeptides (VAP; Synpeptide Biotech Co., Ltd., Nanjing, China) are the main components of velvet antler, and are powerful bioactive substances in them. The velvet antler polypeptide also shows a variety of biological benefits that include anti-osteoporotic, anti-inflammatory, antioxidant, and cell proliferation capabilities, bone density metabolism regulator, and cartilage growth facilitator (Xiao et al., 2017; Wang et al., 2022a; Yao et al., 2021a). Studies have demonstrated that Velvet Antler Polypeptides (VAP) can mitigate chondrocyte damage induced by inflammatory factors, inhibit osteoclast formation and bone resorption, while simultaneously providing protection and repair for cartilage and subchondral bone (Hung et al., 2024; Xia et al., 2022; Ho et al., 2024). However, there is currently no research applying VAP to cartilage tissue engineering. Fibrocartilage stem cells (FCSCs) are found in the condylar apex region of the mandible and have similar properties to mesenchymal stem cells (Embree et al., 2016). FCSCs play an important role in the maintenance of condylar cartilage. Unlike stem cells derived from fibrous connective tissue, FCSCs can spontaneously differentiate into chondrocytes and osteoblasts without external inducible conditions (Nathan et al., 2018; Ko et al., 2020). To date, no studies have explored the role of FCSCs in osteochondral tissue repair.

In this study, we isolated and expanded FCSCs and validated their stem cell properties. We designed a Triphasic scaffold capable of regenerating cartilage and bone defects. In this multi-layer scaffold, layers were designed for cartilage repair, osteochondral isolation and bone repair. The cartilage layer was composed of PLGA/Mg(OH)_2_ with a pore size of 200 μm. The bone layer was made of PLA/PCL with a pore size of 400 μm. A dense PLA/PCL layer was added between the cartilage layer and the bone layer as an intermediate layer to separate the two. Velvet antler polypeptide (VAP) was fixed on the scaffold surface through amide bonds using polydopamine. We evaluated the interaction between the scaffold and FCSCs *in vitro*. FCSCs were inoculated onto the scaffold and their efficacy in treating osteochondral defects was evaluated using SD rats.

## 2 Materials and methods

### 2.1 Fabrication of the triphasic scaffold

We used 3D printing technology to create a three-phase osteochondral scaffold with a 60:40 PLA-PCL weight ratio to achieve the best balance between hydrophilicity and strength. The bone layer and the osteochondral isolation layer were sequentially printed using fused deposition molding (FDM) technology. The cartilage layer was obtained by mixing PLGA and Mg(OH)_2_ at a weight ratio of 85:15 (Park et al., 2018). This mixture is dissolved in methylene chloride and is used for low temperature deposition to create layers of cartilage. The Triphasic osteochondral scaffold was prepared using a 3D printer (Regenovo, Hangzhou, China). The cartilage layer scaffold was manufactured by extrusion 3D printing with a cryogenic nozzle, with the printing temperature set to 10°C, and the aperture set to 200 μm. The bone scaffold layer and the dense bone cartilage isolation layer were 3D printed using an ultra-high temperature nozzle, with the printing temperature set to 170°C, and the design aperture of the bone layer was 400 μm. After printing the 20 mm × 20 mm × 2 mm Triphasic scaffold, the corneal trephine was used to cut it into a scaffold with a thickness of 2 mm and a diameter of 2 mm. Weigh 20 mg of dopamine hydrochloride and dissolve it in 10 mL of Tris-HCl solution (pH = 8.5). Stir the solution magnetically at room temperature in the dark for 15 min, then let it stand for 1 h to allow self-polymerization into polydopamine. Immerse the scaffold in the polydopamine solution for 8 h to ensure surface modification. Dissolve 208 mg of 1-Ethyl-3-(3-dimethylaminopropyl) carbodiimide hydrochloride (EDC; Energy Chemical, Shanghai, China) and 136 mg of N-Hydroxysuccinimide (NHS; Macklin Biochemical Co., Ltd., Shanghai, China) in 10 mL of MES buffer to prepare an activation solution. Weigh 50 mg of VAP and add it to the activation solution. Immerse the polydopamine surface-modified scaffold in the activation solution overnight. After soaking, remove the scaffold and wash it with PBS to obtain the Triphasic PLA/PCL-PLGA/Mg(OH)_2_-VAP Scaffold.

### 2.2 VAP conjugation to scaffolds

The experimental groups consisted of PLA/PCL polydopamine composite scaffolds and PLGA/Mg(OH)_2_ polydopamine composite scaffolds, while the control groups were made up of PLGA/Mg(OH)_2_ composite scaffolds and PLA/PCL composite scaffolds. Dissolve Fluorescein isothiocyanate (FITC; Abbkine Scientific Co., Ltd., Wuhan, China) and VAP in DMSO and stir the solution in the dark for 8 h to obtain the reaction mixture. Transfer this mixture into a dialysis bag with a 1,000 Da molecular weight cut-off and perform dialysis in the dark for 3 days. Afterward, transfer the solution from the dialysis bag to a centrifuge tube, keep it in the dark, and freeze it at −80°C overnight. Move the frozen solution to a lyophilizer and freeze-dry for 3 days to obtain the FITC-VAP conjugate. Place FITC-VAP in a prepared activation solution, and react it with the scaffold surface in the presence of NHS and EDC to form amide bonds, thereby immobilizing FITC-VAP on the polydopamine-modified scaffold surface. Finally, we applied confocal microscopy to ensure the successful attachment of FITC-VAP.

### 2.3 Characterization of triphasic scaffolds

#### 2.3.1 Microstructural characterization of triphasic scaffolds

The fabricated triphasic PLA/PCL-PLGA/Mg(OH)_2_-VAP scaffold was examined using scanning electron microscopy (SEM) to determine its microstructure and pore size. An SEM device (Japan Electronics Laboratory, Tokyo, Japan), which can be operated at an accelerating voltage of 10.0 kV, was used for the observations. Pore formation and structure on the top surfaces, bottom surfaces, and cross-sections (transversal slices) of the scaffolds were studied.

#### 2.3.2 Mechanical characterization of scaffolds

Compression testing was performed on the bone layer scaffold: PLA/PCL-VAP, and the cartilage layer scaffold: PLGA/PLGA/Mg(OH)_2_-VAP, using a universal testing machine (CMT6103, MTS Systems, United States). A compression rate of 1 mm/min was applied, and the force-displacement data were recorded and converted into stress-strain curves. The compressive modulus was calculated from the linear region of these curves. Data analysis was conducted using Origin software (OriginLab, United States). All tests were carried out at room temperature, and the results are reported as mean ± standard deviation (n = 3).

#### 2.3.3 Energy spectrum and energy dispersive X-ray spectroscopy (EDS) mapping of the scaffolds

To determine the distribution of magnesium in the scaffold, EDS analysis and mapping were performed on the cartilage layer: PLGA/Mg(OH)_2_-VAP scaffold using an EDS system connected to a SEM (Gemini 300, Carl Zeiss).

#### 2.3.4 X-ray diffraction (XRD) analysis

The crystalline structure of scaffold components (PCL, PLA, PLGA, Mg(OH)_2_, VAP), as well as bone layer scaffolds PLA/PCL, PLA/PCL-PDA, PLA/PCL-VAP, and cartilage layer scaffolds PLGA/Mg(OH)_2_, PLGA/Mg(OH)_2_-PDA, PLGA/Mg(OH)_2_-VAP, was examined using an XRD (Anton Paar GmbH, Graz, Austria). Diffraction patterns were recorded over a 2θ range of 10°–80° at a scan speed of 2°/min. The resulting diffraction patterns were analyzed.

#### 2.3.5 Fourier transform infrared spectroscopy (FTIR) analysis

The chemical composition and functional groups of scaffold components (PCL, PLA, PLGA, Mg(OH)_2_, VAP), as well as bone layer scaffolds PLA/PCL, PLA/PCL-PDA, PLA/PCL-VAP, and cartilage layer scaffolds PLGA/Mg(OH)_2_, PLGA/Mg(OH)_2_-PDA, PLGA/Mg(OH)_2_-VAP, were examined using a Fourier-transform infrared spectrometer (Bruker Optics GmbH, Ettlingen, Germany). Spectra were recorded in the range of 4,000–400 cm⁻^1^, with data acquisition and analysis conducted using OPUS software.

### 2.4 Culture and biological characteristics of FCSCs

#### 2.4.1 Isolation and in vitro culture of FCSCs

Under sterile conditions, condyles were harvested from 3-week-old male rats. The fibrous tissues of the superficial layers of the condyles were dissected, minced, and incubated in a 0.3% type I collagenase and 0.4% neutral protease solution at 37°C for 30 min. The enzymatic activity was halted by adding low-glucose Dulbecco’s Modified Eagle’s Medium (DMEM; Gibco, Grand Island, New York, United States) supplemented with 20% fetal bovine serum (FBS; Gibco, Grand Island, New York, United States). The cell suspension was centrifuged, and the obtained cells were cultured in DMEM with 20% FBS, 100 U/mL penicillin (Gibco, Grand Island, New York, United States), and 100 U/mL streptomycin (Gibco, Grand Island, New York, United States) at 37°C, with medium changes every 2 days. When the cells reached 80%–90% confluence, they were passaged using a 0.25% trypsin/ethylenediaminetetraacetic acid (EDTA) solution.

#### 2.4.2 Flow cytometry analysis

Single-cell suspensions were incubated with or without fluorochrome-conjugated specific antibodies at the recommended concentrations for 30 min at 4°C. Subsequently, the cells were washed and resuspended in phosphate-buffered saline (PBS) for analysis using a flow cytometer (BD Biosciences, Franklin Lakes, New Jersey, United States). Unstained cells served as blank controls. The antibodies used for staining included APC-conjugated anti-rat CD29 (BioLegend, San Diego, California, United States), CD45 (BD Biosciences), and CD11B (BioLegend, San Diego, California, United States), as well as PE-conjugated anti-rat CD44 (BioLegend, San Diego, California, United States), CD79a (BD Biosciences, Franklin Lakes, New Jersey, United States), and CD90 (BD Biosciences, Franklin Lakes, New Jersey, United States). The stained cells were analyzed using the APC and PE channels on the flow cytometer.

#### 2.4.3 *In Vitro* multilineage differentiation and spontaneous differentiation assays

The multilineage differentiation potential of the rat FCSCs was assessed *in vitro* using corresponding lineage-specific induction media for chondrogenesis, osteogenesis, and adipogenesis. For chondrogenic differentiation, FCSCs (1 × 10*6) were cultured in 6-well plates with a complete chondrogenic induction medium (Cyagen, Guangzhou, China) for 3 weeks. Alcian blue staining was performed to visualize the cartilaginous matrix. To induce osteogenic differentiation, FCSCs (1 × 10*6) were cultured in 6-well plates with a complete osteogenic induction medium (Cyagen, Guangzhou, China) for 3 weeks. Alizarin red staining was used to observe calcium nodules. For adipogenic induction, FCSCs (1 × 10*6) were cultured in 6-well plates with a complete adipogenic induction medium (Cyagen, Guangzhou, China) for 3 weeks. Oil Red O staining was employed to detect lipid droplets. FCSCs (1 × 10*6) were pelleted in 15 mL polypropylene tubes via centrifugation at 1,000 rpm for 5 min. The cell pellets were then cultured in a complete chondrogenic induction medium (Cyagen, Guangzhou, China) for 3 weeks, with medium changes every 2 days. After 3 weeks, the cell pellets were processed for histological analysis, and Alcian blue staining was performed to evaluate the presence of cartilaginous matrix.

### 2.5 Preparation of scaffold extracts

Sample extraction was performed according to the Chinese National Standard for Biological Evaluation of Medical Devices (GB/T 16886.12–2005/ISO 10993–12:2002). Extracts of the three-phase PLA/PCL-PLGA/Mg(OH)_2_-polydopamine and PLA/PCL-PLGA/Mg(OH)_2_-VAP three-phase scaffolds were prepared using low-glucose Dulbecco’s Modified Eagle Medium (DMEM; Gibco, Grand Island, New York, United States), which is a complete culture medium. Extracts of the PLA/PCL-polydopamine and PLA/PCL-VAP scaffolds were prepared using a complete osteogenic induction medium (Cyagen, Guangzhou, China), while extracts of the PLGA/Mg(OH)_2_-polydopamine and PLGA/Mg(OH)_2_-VAP scaffolds were prepared using a complete chondrogenic induction medium(Cyagen, Guangzhou, China). The scaffold materials were sterilized via ultraviolet irradiation, and extracts were prepared at a concentration of 0.1 g/mL. The extracts were incubated at 37°C in a 5% CO2 incubator for 24 h and then sterilized via filtration through a microporous membrane. The resulting extracts were sealed in sterile bottles and stored in a refrigerator at 4°C.

### 2.6 Cell proliferation and viability

FCSCs’ proliferation was measured using the CCK-8 assay. FCSCs were implanted in the supernatant from the extracts of the three-phase poly(lactic acid)/poly(caprolactone) and lactic acid)/poly(lactide-co-glycolide)/Mg(OH)_2_-polydopamine scaffolds (Control) and three-phase PLA/PCL-PLGA/Mg(OH)_2_-VAP scaffolds (VAP-Scaffold). The blank group was the normal low-glucose DMEM complete culture medium for 1, 3, 5, and 7 days. Consequently, 100 μL of the CCK-8 detection reagent was added to each well of the plate, and cells under study were cultured at 37°C for 1 h. The absorption was measured using an ELISA reader (Biobase, Qingdao, China). By measuring the optical density (OD) values, the number of living cells could be calculated as this parameter is directly proportional to cell number. Thus, OD values were linked to the proliferation of cultivated FCSCs and used to monitor the results. Additionally, a live/dead assay was implemented to reveal the cell distribution and viability of cells on the scaffolds. The FCSCs were incubated onto the corresponding scaffolds and cultured for 1, 3, 5, and 7 days. Afterwards, they were stained using a two-color kit containing calcein-AM (Solarbio, Beijing, China) for bright green (live cells) and propidium iodide (Solarbio, Beijing, China) for dull red (dead cells).

### 2.7 Osteogenic differentiation and mineralization assays

Third-passage FCSCs were seeded at a density of 1 × 10^6 cells/cm^2 in 6-well plates (Corning, New York, United States). After 24 h, the original culture medium was replaced with either a normal complete medium for the induction of osteogenic differentiation (Blank) or a complete medium for the induction of osteogenic differentiation containing extracts from PLA/PCL-polydopamine scaffolds (Control) or PLA/PCL-VAP scaffolds (VAP-Scaffold). The culture medium was changed every 3 days. After 14 days, the FCSC alkaline phosphatase (ALP) activity was measured using a commercial assay kit (Cyagen, Guangzhou, China). Following 21 days of culture, the mineralized matrix deposition was assessed by alizarin red staining for calcium.

### 2.8 Chondrogenic differentiation assays

FCSCs were seeded at 1 × 10^6 cells/cm^2 in 6-well plates. After 24 h, the original medium was replaced with either a normal complete medium (Blank) or a complete medium containing extracts from PLGA/Mg(OH)_2_-polydopamine (Control) or PLGA/Mg(OH)_2_-VAP (VAP-Scaffold) scaffolds, to induce chondrogenic differentiation. The medium was changed every 3 days. After 21 days of incubation, cartilage matrix formation was evaluated using Alcian blue staining.

### 2.9 Western blotting

Proteins were extracted from FCSCs using RIPA lysis buffer supplemented with protease inhibitors (Thermo Fisher Scientific, Waltham, MA, United States). The protein concentrations were determined using the Pierce BCA Protein Assay Kit (Thermo Fisher Scientific). Equal amounts of protein samples were separated by SDS-PAGE and transferred onto PVDF membranes (Millipore, Billerica, MA, United States). The membranes were blocked with 5% Bovine Serum Albumin (BSA, Sigma-Aldrich) at room temperature for 1 h, and then incubated overnight at 4°C with primary antibodies against COL2 (1:1,000, Abcam), SOX9 (1:200, Santa Cruz Biotechnology), RUNX2 (1:500, Cell Signaling Technology), and Osterix (1:1,000, Novus Biologicals).

This was followed by incubation with HRP-conjugated secondary antibodies (1:5,000, Jackson ImmunoResearch) for 1 h at room temperature. Protein bands were visualized using an Enhanced Chemiluminescence (ECL) detection system (Bio-Rad) and quantified by densitometry analysis using Image Lab™ software (version 4.0, Bio-Rad). β-actin (1:10000, Sigma-Aldrich) served as the loading control.

### 2.10 *In Vivo* osteochondral defect repair

The cell suspension was placed directly into the scaffolds, and the complexes were cultured in a cell incubator with 5% CO2 at 37°C to allow rapid cell expansion on the scaffolds.

All the experiments were approved by the Animal Research Ethics Committee of Kunming Medical University (Ethics approval number: KMMU20211,538). A group of 12-week-old male SD rats weighing 380 g ± 25 g were used in this study. The rats were first anesthetized with 1% pentobarbital sodium (40 mg/kg), shaved, and disinfected. The knee joint was exposed using a medial parapatellar approach, and the patella was dislocated laterally. Cylindrical osteochondral defects with 2 mm diameters and depths were created at the center of the trochlear grooves of both hind limbs using a dental drill (Meng et al., 2020; Wang et al., 2022b). All the debris in the defect area was removed with a curette and irrigation. A series of scaffolds were implanted into the defect area via press-fit: (1) a three-phase PLA/PCL-PLGA/Mg(OH)_2_-polydopamine scaffold (Scaffold), (2) a three-phase PLA/PCL-PLGA/Mg(OH)_2_-VAP scaffold (VAP-Scaffold), and (3) a three-phase PLA/PCL-PLGA/Mg(OH)_2_-VAP scaffold loaded with FCSCs (VAP-Scaffold-FCSCs); (n = 4 per group). A group of rats without scaffold implantation served as the blank group (Blank). At 8 weeks post-surgery, the rats were euthanized, and the heart, liver, spleen, lungs, and kidneys were harvested for H&E staining. The femurs of the rats were harvested, scanned using Micro-CT (Pingseng Scientific, Suzhou, China), and analyzed and reconstructed using Mimics software. The samples were decalcified and processed for paraffin sectioning. Safranin O/Fast Green and toluidine blue staining were performed to evaluate new cartilage and bone formation. Cartilage regeneration was assessed according to the International Cartilage Repair Society (ICRS) scoring system (Table 1).

**TABLE 1:** Cartilage repair assessment (ICRS).

Scoring items	Index parameters	Score
Degree of Defect Repair	Complete repair of defect depth	4
	75% repair of defect depth	3
	50% repair of defect depth	2
	25% repair of defect depth	1
	0% repair of defect depth	0
Integration in Border Zone	Complete integration with surrounding cartilage	4
	Demarcating border <1 mm	3
	3/4 of graft integrated; and 1/4 with a notable border >1 mm wide	2
	1/2 of graft integrated with surrounding cartilage; and 1/2 with a notable border >1 mm	1
	From no contact to 1/4 of graft integrated with surrounding cartilage	0
Macroscopic Appearance	Intact smooth surface	4
	Fibrillated surface	3
	Small, scattered fissures or cracks	2
	Several; small fissures or a few large fissures	1
	Total degeneration of grafted area	0
Overall Repair Assessment	Grade I: normal	12
	Grade II: nearly normal	11–8
	Grade III: abnormal	7–4
	Grade IV: severely abnormal	3–1

### 2.11 Statistical analysis

Statistical analysis was performed with SPSS 26.0, and quantitative data are described using medians and interquartile ranges. Nonparametric tests were used for intergroup mean comparisons and multiple comparisons. Bar graphs were created with GraphPad Prism 9. A *p*-value below 0.05 was considered statistically significant.

## 3 Results and discussion

### 3.1 Fabrication and characterization of triphasic PLA/PCL-PLGA/Mg(OH)_2_-VAP scaffolds

**FIGURE 1:**
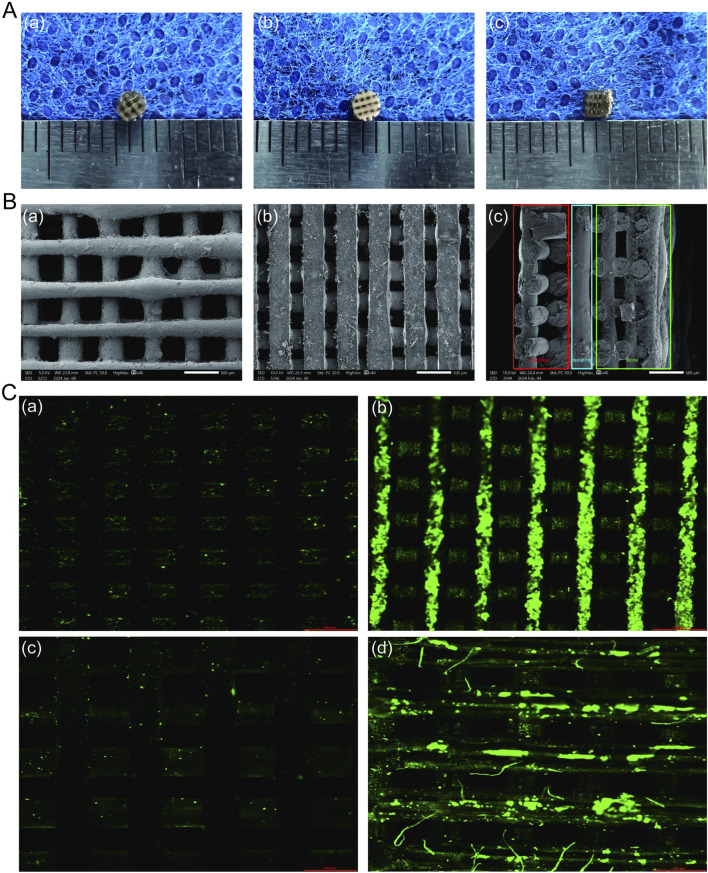
Characterization of PLA/PCL-PLGA/Mg(OH)_2_-VAP composite scaffolds. **(A)** Gross observations: **(A)** surface of the bone layer, **(B)** surface of the cartilage layer, **(C)** lateral view showing the layered structure (scale bar = 1 mm). **(B)** Scanning electron microscopy: **(A)** bone layer surface with ∼400 μm pores, **(B)** cartilage layer surface with ∼200 μm pores, **(C)** cross-sectional view of the layered structure (scale bar = 500 μm). Red: bone layer; Green: cartilage layer; Blue: isolation layer. **(C)** Confocal microscopy of VAP attachment: **(A)** PLA/PCL, **(B)** PLA/PC-polydopamine L, **(C)** PLGA/Mg(OH)_2_, **(D)** PLGA/Mg(OH)_2_-polydopamine. Scaffolds in **(B)** and **(D)** were pre-treated with polydopamine. Green fluorescence indicates FITC-labeled VAP (scale bar = 500 μm).

The successful fabrication of triphasic PLA/PCL-PLGA/Mg(OH)_2_-VAP scaffolds was confirmed via visual inspection and scanning electron microscopy (SEM). As depicted in Figure 1A, the scaffolds displayed a uniform cylindrical shape with a diameter and thickness of about 2 mm. Cross-sectional SEM images (Figure 1B) revealed a distinct triphasic layered structure, with each layer exhibiting a highly porous architecture. The cartilage layer scaffold, containing Mg(OH)_2_, displayed a pore size of approximately 200 μm, while the bone layer scaffold had a larger pore size of around 400 μm. The intermediate layer, which comprises PLA/PCL, serves as the interface between the cartilage and bone layers, leaving these outer layers in isolation.

**FIGURE 2:**
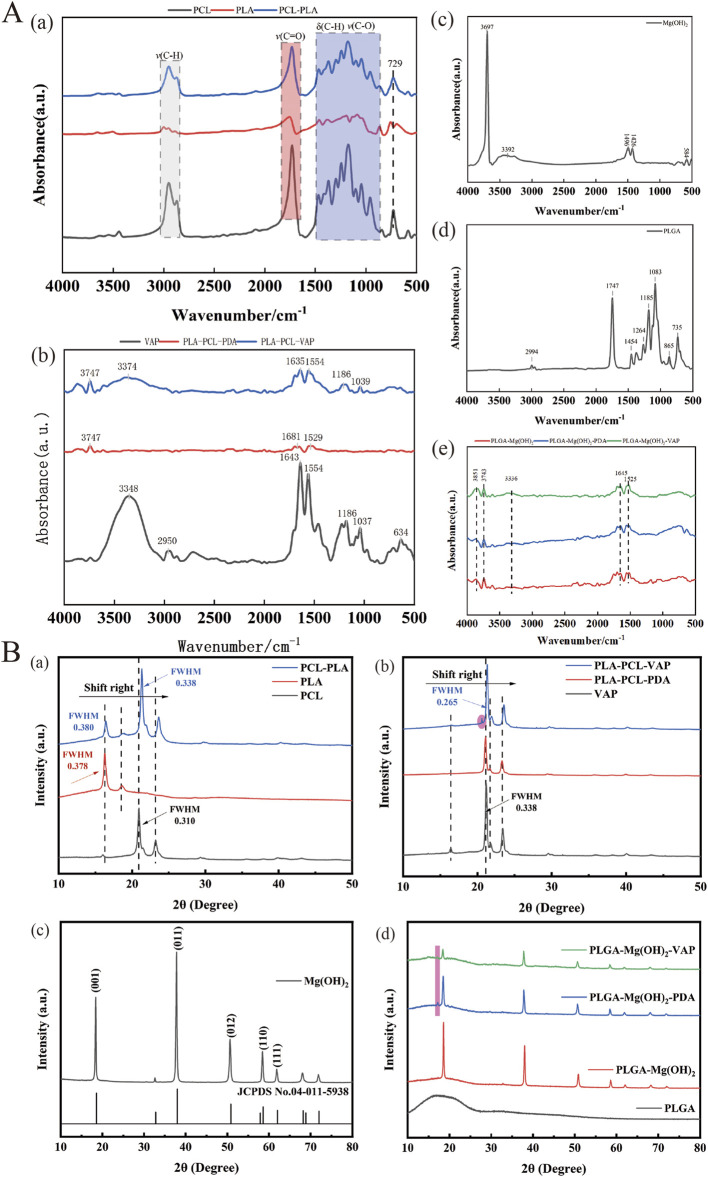
Components of the triphasic PLA/PCL-PLGA/Mg(OH)_2_-VAP scaffold.**(A)** FTIR spectra of the scaffold components. **(B)** XRD profiles of the scaffold components.

The introduction of VAP into the matrix was observed using confocal microscopy (Figure 1C). The surface properties of the scaffold were modulated through the infiltration of VAP molecules. These molecules can bind to the scaffold surface via amide bonds with polydopamine. Similarly, FTIR analysis of PCL, PLA, and their composites reveals characteristic peaks at 2,956 cm⁻^1^, 1730 cm⁻^1^, and 1,458–1,171 cm⁻^1^, attributed to C-H and C-O vibrations. The PCL/PLA spectrum suggests physical interactions between polymers, while PCL/PLA-PDA shows significant changes. VAP’s spectrum exhibits distinct peaks, notably at 3,348 cm⁻^1^ (N-H stretching) and 1,643 cm⁻^1^ (C=O stretching). PCL/PLA-VAP shows a new peak at 3,374 cm⁻^1^, indicating successful VAP grafting onto PCL/PLA-PDA, with evidence of hydrogen bonding. Mg(OH)₂ spectrum shows characteristic peaks at 3,697 cm⁻^1^ and 3,392 cm⁻^1^ (O-H stretching), while PLGA exhibits key peaks at 2,994 cm⁻^1^ (C-H stretching) and 1747 cm⁻^1^ (C=O stretching). Comparison of PLGA/Mg(OH)₂, PLGA/Mg(OH)₂-PDA, and PLGA/Mg(OH)₂-VAP spectra reveals that PDA introduction does not significantly alter the chemical structure, while a new N-H stretching peak at 3,336 cm⁻^1^ in PLGA/Mg(OH)₂-VAP confirms successful (Figure 2A). VAP incorporation. XRD analysis reveals characteristic peaks for PCL (21.4°, 23.7°) and PLA (16.7°, 19.1°). The PLA/PCL blend shows peaks from both components with slight shifts to higher angles and increased FWHM. PLA/PCL-PDA exhibits significant changes, with PLA peaks disappearing and the strongest peak shifting to 21.053°. VAP’s pattern is similar to PLA/PCL-PDA but with a distinct peak at 16.473°. In PLA/PCL-VAP, this peak disappears, and a new peak emerges at 20.586°, indicating successful VAP grafting. Mg(OH)₂ shows characteristic peaks at 18.358°, 37.765°, 50.598°, 58.419°, and 61.854°. PLGA/Mg(OH)₂ exhibits peak shifts and changes in relative intensities. PLGA/Mg(OH)₂-PDA shows a new peak at 17.201°, confirming PDA grafting. PLGA/Mg(OH)₂-VAP’s pattern resembles original Mg(OH)₂ more closely, with the 17.201° peak persisting, indicating successful VAP incorporation (Figure 2B). Therefore, the chemical linkage of VAP provides sufficient stability for the sustained release of bioactive compounds in the body.

**FIGURE 3:**
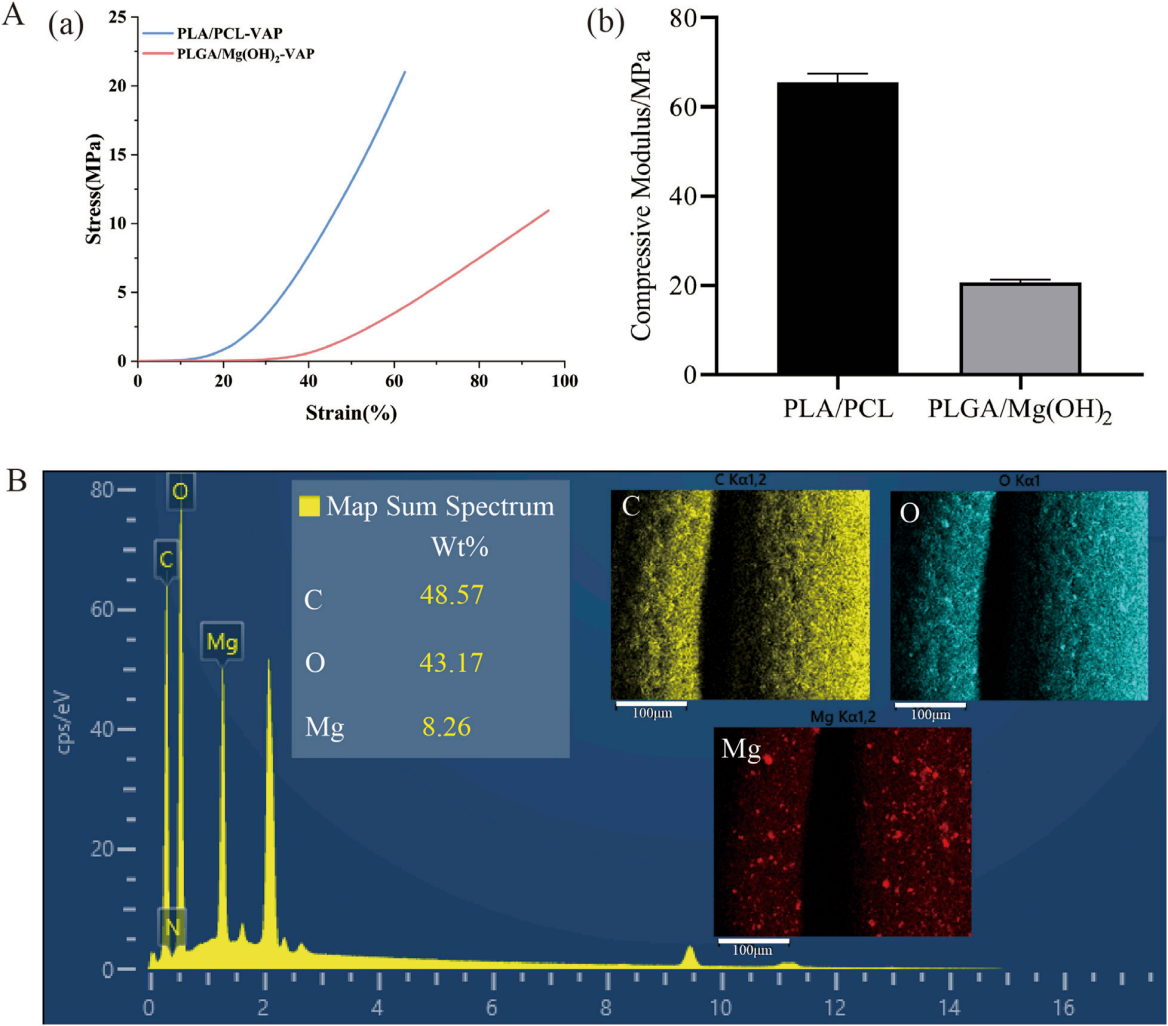
Mechanical properties and elemental analysis of the layered scaffold. **(A)** Mechanical characterization: **(A)** Stress-strain curves of the cartilage and bone layers under compression, **(B)** Compressive moduli of the cartilage and bone layers (mean ± SD, n = 3). **(B)** Elemental analysis: Energy dispersive X-ray spectroscopy (EDS) spectrum of the scaffold, EDS mapping showing the distribution and proportion of carbon (C), oxygen (O), and magnesium (Mg) (scale bar = 100 μm).

The layered scaffold developed in this study exhibits stratified mechanical properties similar to natural osteochondral tissue. The stress-strain curves of the cartilage and bone layers are shown in Figure 3Aa, with different compressive moduli depicted in Figure 3Ab. The compressive modulus of the cartilage layer is 20.7086 ± 0.63024 MPa, while the bone layer has a compressive modulus of approximately 65.5044 ± 1.98451 MPa (Supplementary Table S1). This gradient structure effectively simulates the mechanical characteristics of different regions within osteochondral tissue (Nooeaid et al., 2012). The lower compressive modulus of the cartilage layer allows it to undergo greater deformation, better mimicking the cushioning and shock-absorbing functions of cartilage. The compressive modulus of the bone layer falls within the range of natural cancellous bone (50–500 MPa), providing the necessary support for the overall structure and demonstrating good biomechanical compatibility (Hutmacher et al., 2007). This design, which mimics the structure of natural tissue, holds promise for achieving better reconstructive outcomes in the repair of osteochondral defects. The energy spectrum and EDS mapping show that the elements C, O, and Magnesium (Mg) are evenly distributed on the scaffold (Figure 3B). Mg-based materials can promote cartilage differentiation(Buckley et al., 2024; Rasoulianboroujeni et al., 2018), so the cartilage layer scaffold in this study can provide a uniform cartilage modulating effect.

The design of the triphasic scaffolds is based on their porous structure, which is a crucial factor for promoting cellular adhesion, proliferation, and differentiation. The size of the pores is optimal for the cartilage layer, at 200 μm, and for the bone layer, at 400 μm, which aligns closely with literature values for promoting proper chondrogenesis and osteogenesis (Yang et al., 2023; Duan et al., 2014). The porous interconnected network also aids in the efficient transfer of nutrients and the removal of waste (Loh and Choong, 2013; Rasoulianboroujeni et al., 2018; Buckley et al., 2024). Earlier studies have already established that such minerals can enhance chondrogenesis, as was observed in the promotion of cartilage differentiation by magnesium-based materials (Hu et al., 2018; Yao et al., 2021b; Li et al., 2024). This mineral may confer additional chondrogenic ability to the final tissue-engineered scaffold if it is indeed located in the cartilage zone.

### 3.2 Identification and characterization of FCSCs

**FIGURE 4:**
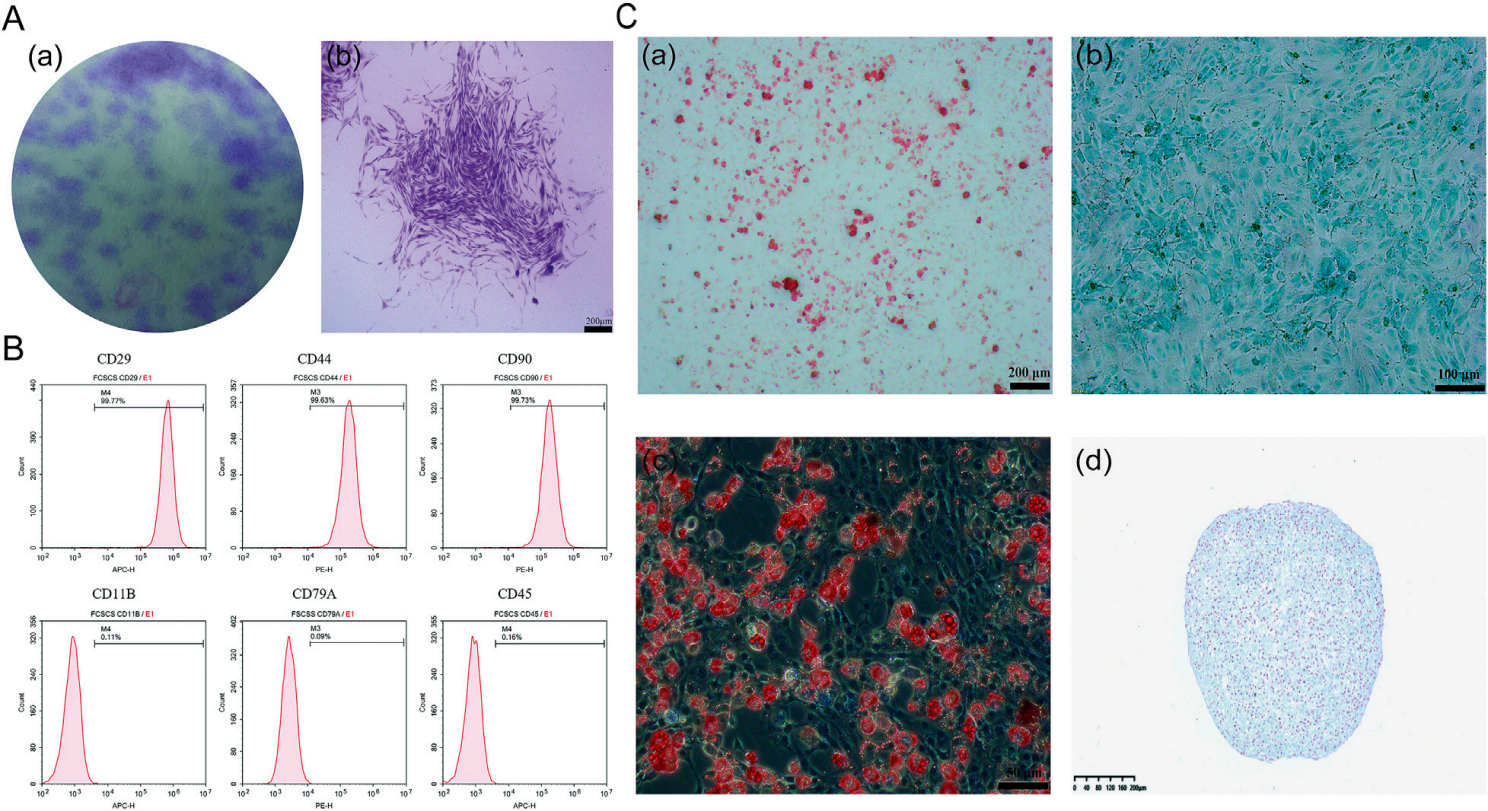
Characterization and differentiation potential of FCSCs. **(A)** Crystal violet staining of FCSCs in separate images: **(A)** Optical view showing purple areas, which are the individual clonal colonies; **(B)** morphology of the FCSC clonal clusters showing branches of the cells (scale bar = 200 µm). **(B)** Flow cytometry analysis: FCSCs were positive for CD29, CD90, and CD44 but negative for CD11B, CD79A, and CD45. **(C)** Trilineage differentiation and three-dimensional culture: **(A)** osteogenic differentiation cells stained with Alizarin Red S (scale bar = 200 µm); **(B)** For chondrogenic differentiation, cells stained with Alcian blue (scale bar = 100 µm); **(C)** Cells stained with Oil Red O, indicating the presence of lipid droplets representing adipogenesis (scale bar = 50 µm); **(D)** In the cartilage-like spheroids (CLS) derived from FCSCs in three-dimensional culture, cells were stained with Alcian blue, resulting in blue coloration, showing the presence of a proteoglycan matrix (scale bar = 200 µm).

Fibrocartilage stem cells (FCSCs) were successfully isolated from the superficial layer of rat condylar fibrocartilage and used to generate single-cell colonies. The isolated cells formed adherent clones of fibroblast-like cells, creating distinct cell colonies (Figure 4A). Similar findings were seen in research, as the information obtained from previous research discovered the fact that stem cells exist in the fibrous layer located at the side of the condyle (Embree et al., 2016).

To further characterize the isolated FCSCs, flow cytometry was performed to examine surface marker expression. As illustrated in Figure 4B, the FCSCs were positive for CD29, CD90, and CD44, and negative for CD11B, CD79A, and CD45. The molecular footprint of FCSCs was as previously described (Embree et al., 2016). The profile of CD29, CD90, and CD44 expression denotes the cells as MSCs (mesenchymal stem cells), as these markers have been frequently found in different kinds of MSC groups such as bone marrow, synovial, and adipose stem cells too (Fellows et al., 2016, *p* et al., 2011; Suto et al., 2017). The lack of CD11B, CD79A, and CD45 markers demonstrated that the FCSCs were not immune cells, which is dissimilar from the cells of the immune system. These findings suggest that FCSC may be mesenchymal stem cells. (Fonseca et al., 2023; Ayyanar et al., 2021).

The multidirectional differentiation potential of FCSCs was confirmed through staining after trilineage differentiation induction (Figure 4C), indicating that these FCSCs are indeed capable of differentiating into cartilage, bone, and adipose tissue, respectively. To further confirm the chondrogenic potential of FCSCs, they were cultured in three dimensions, and it was found that they could spontaneously form cartilage-like spheroidal structures. It was demonstrated that FCSCs have the ability to spontaneously differentiate into cartilage. This finding supports the notion that FCSCs may be a superior choice as seed cells for osteochondral tissue engineering.

### 3.3 Biocompatibility of triphasic PLA/PCL-PLGA/Mg(OH)_2_-VAP scaffold

**FIGURE 5:**
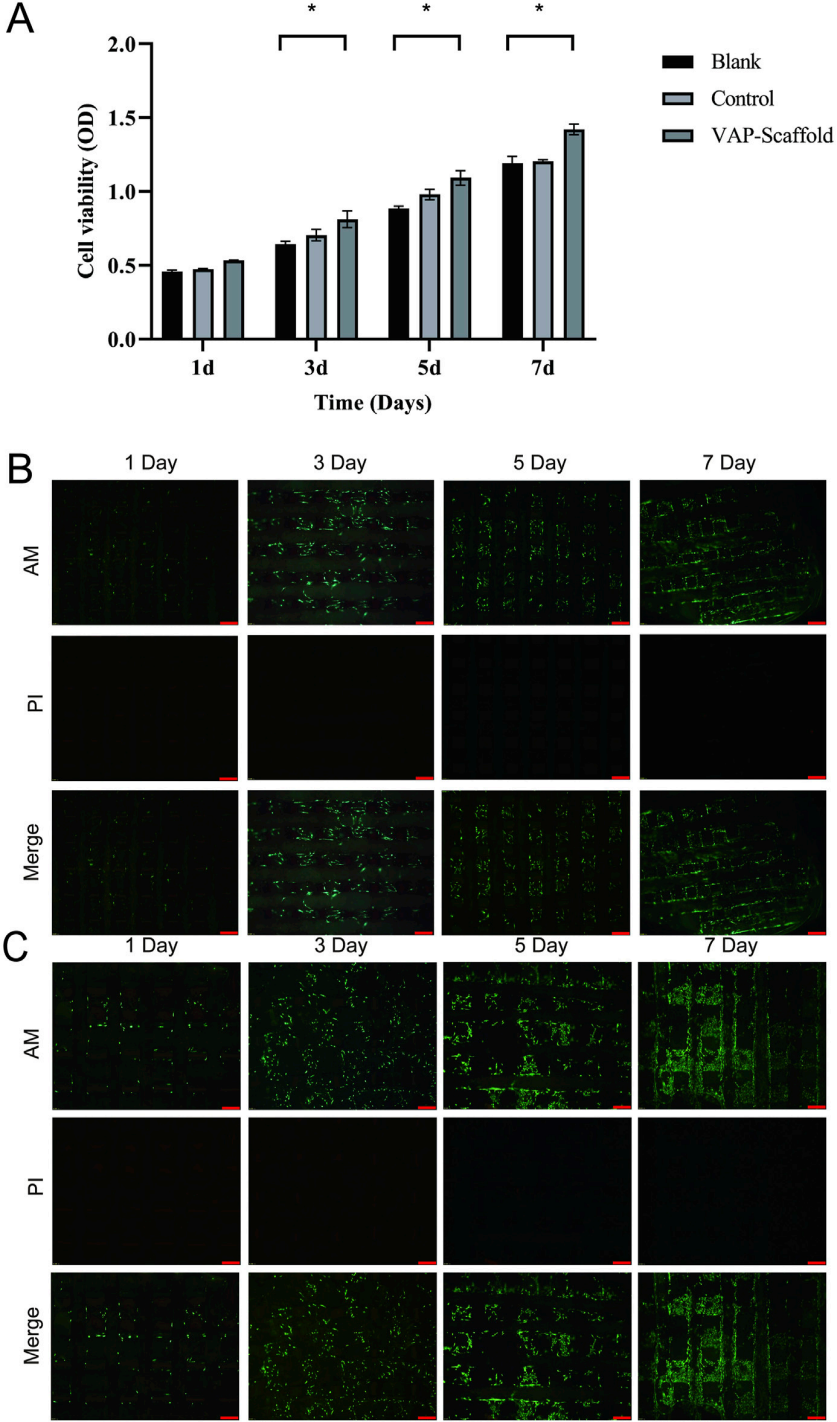
Cell viability assessment and Live/Dead staining analysis of composite scaffolds. **(A)** Results are presented as a bar graph from the CCK-8 assay, showing cell viability on each designated day (1, 3, 5, and 7). Asterisks (*) indicate statistically significant differences (*p* < 0.05). **(B)** Live/Dead staining of PLA/PCL-VAP composite scaffold imaged by inverted fluorescence microscopy. Viable cells are labeled with green fluorescence (AM staining), while dead cells are observed in red (PI staining). The merged image shows the distribution of live and dead cells. Scale bar: 200 µm. **(C)** Live/Dead staining of PLGA/Mg(OH)_2_-VAP composite scaffold imaged by inverted fluorescence microscopy. AM staining labeled the live cells green, while the dead cells were red (PI staining), and the overlaid images demonstrated the combined live and dead cells. Scale bar: 200 µm.

In the field of tissue engineering, the use of biomaterial scaffolds with good biocompatibility is crucial for promoting the repair and regeneration of damaged tissues. This study employed a CCK-8 assay to evaluate the cytotoxicity of triphasic PLA/PCL-PLGA/Mg(OH)_2_-VAP scaffolds on fibrocartilage stem cells (FCSCs) (Figure 5A). The CCK-8 assay is a commonly used method for assessing cell proliferation and cellular activity (Cai et al., 2019). The FCSCs exhibited a significantly increasing rate in all the experimental groups. The findings reveal that the presence of Mg(OH)_2_-VAP gave the tri-phasic PLA/PCL-PLGA-Mg(OH)_2_-VAP scaffold the ability to significantly elevate FCSCs’ proliferation on days 3, 5, and seven compared with the controls (*p* < 0.05). FCSC growth on the scaffold was further evidenced in live/dead cell staining experiments, as described by Podgorski (Podgorski et al., 2022). FCSCs were cultured in the PLA/PCL-VAP and PLGA/Mg(OH)_2_-VAP composite scaffolds and were found to express green fluorescence in assays. Moreover, minimal red fluorescence accompanied by dead cells was noted at all time points (Figures 5B,C), signifying that FCSCs indeed grew well. Hence, it may be concluded that these scaffold materials exhibited good biocompatibility, and this helped in the normal attachment and growth of the cells on the tri-phasic scaffold.

Our findings indicate that the tri-phasic PLA/PCL-PLGA/Mg(OH)_2_-VAP scaffold enhances FCSC activity. This enhancement may be attributed to the incorporation of VAP, which has been shown to promote cell growth and differentiation in previous studies (Xiao et al., 2017; Wang et al., 2022a; Yao et al., 2021a).

### 3.4 Osteogenic and chondrogenic differentiation of FCSCs on scaffolds

We investigated the effects of triphasic PLA/PCL-PLGA/Mg(OH)_2_-VAP scaffold compositions on the osteogenic and chondrogenic differentiation potentials of Fibrocartilage Stem Cells (FCSCs), highlighting the role of VAP.

**FIGURE 6:**
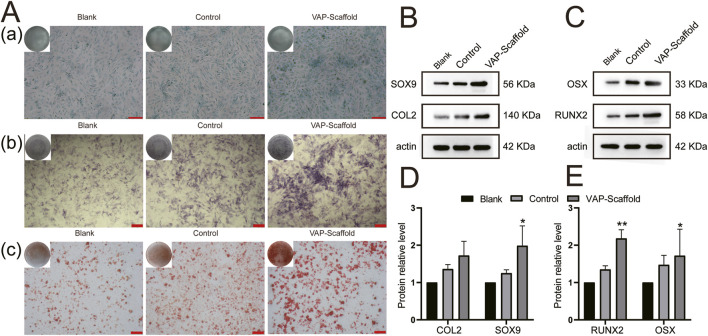
Osteogenic and Chondrogenic Differentiation Analysis. **(A)** Osteogenic and chondrogenic staining results: **(A)** Alcian blue staining after 21 days of chondrogenic induction; (scale bar = 100 µm). **(B)** ALP staining after 14 days of osteogenic induction; (scale bar = 200 µm). **(C)** Alizarin Red S staining after 21 days of osteogenic induction; (scale bar = 200 µm). **(B)** Chondrogenic protein expression levels **(C)** Osteogenic protein expression levels **(D)** Chondrogenic protein expression quantitative analysis **(E)** Osteogenic protein expression quantitative analysis. * and ** representing *p* < 0.01 and *p* < 0.001 respectively.

The assessment of osteogenic differentiation revealed that, after 14 days of induction, the VAP-Scaffold group exhibited a significant increase in Alkaline Phosphatase (ALP) activity. ALP is a recognized early marker of osteogenic differentiation, and its increased activity indicates active bone formation (Trivedi et al., 2020). During osteogenesis, ALP promotes bone mineralization by hydrolyzing phosphate esters to provide inorganic phosphate (Ansari et al., 2022). Alizarin Red S is a dye widely used to assess *in vitro* mineralization and bone formation by forming a bright red precipitate with calcium ions to indicate the presence of calcium (Gregory et al., 2004). After 21 days, Alizarin Red S staining further demonstrated a marked increase in calcium deposition within the VAP-Scaffold group (Figure 6A). These findings are consistent with a study by Li (Yun et al., 2020), which demonstrated that VAP significantly promotes osteogenic differentiation by enhancing cell proliferation and mineralization.

Runx2 and Osterix are key transcription factors in osteogenesis. Runx2 is a primary regulator in the differentiation of mesenchymal stem cells into osteoblasts (Zuo et al., 2012). Osterix functions downstream of Runx2, and studies have shown that mice lacking Osterix fail to form bone tissue, highlighting its importance in bone formation (Nakashima et al., 2002). Western blot analysis revealed that, after 21 days of osteogenic induction, the expression levels of Runx2 and Osterix were significantly higher in the VAP-Scaffold group compared to the control and Blank-Scaffold groups (Figures 6C,E), providing molecular-level evidence for the osteoinductive properties of VAP-enhanced scaffolds.

In terms of chondrogenic differentiation, Alcian Blue staining is widely used to detect glycosaminoglycans (GAGs) in cartilage tissue. SOX9 and COL2 are key markers in cartilage formation. SOX9 is a critical transcription factor that plays a decisive role in the early differentiation of mesenchymal stem cells into chondrocytes and regulates the expression of several cartilage-specific genes, including the COL2A1 gene encoding COL2 (Liu and Lefebvre, 2015). COL2 is a major structural protein in the cartilage extracellular matrix and is considered a marker of mature cartilage tissue (Sophia Fox et al., 2009). Therefore, the expression levels of SOX9 and COL2 together reflect the extent of chondrogenic differentiation. after 21 days of induction, the most intense Alcian blue staining was observed in the VAP-Scaffold group (Figure 6A), which aligns with the findings of Yao et al. (Yao et al., 2021a), regarding VAP’s role in promoting chondrogenic differentiation. Concurrently, Western blot analysis revealed a significant increase in the expression of Sox9 and Col2 within the VAP-Scaffold group (Figures 6B,D), further corroborating the enhanced chondrogenic differentiation potential of VAP-augmented scaffolds.

The simultaneous upregulation of both osteogenic markers (Runx2,Osterix) and chondrogenic markers (Sox9,Col2) in the VAP-Scaffold group suggests that this composite scaffold may provide a conducive microenvironment for the differentiation of FCSCs into both bone and cartilage lineages. These findings underscore the potential application of VAP-enhanced scaffolds in the regeneration of complex tissues, especially those requiring multi-lineage stem cell differentiation. The enhancement of both osteogenic and chondrogenic differentiation in the presence of VAP suggests a possible synergistic effect between the scaffold composition and the bioactive peptide.

### 3.5 Systemic toxicity evaluation and in vivo assessment of scaffold treatment efficacy

**FIGURE 7:**
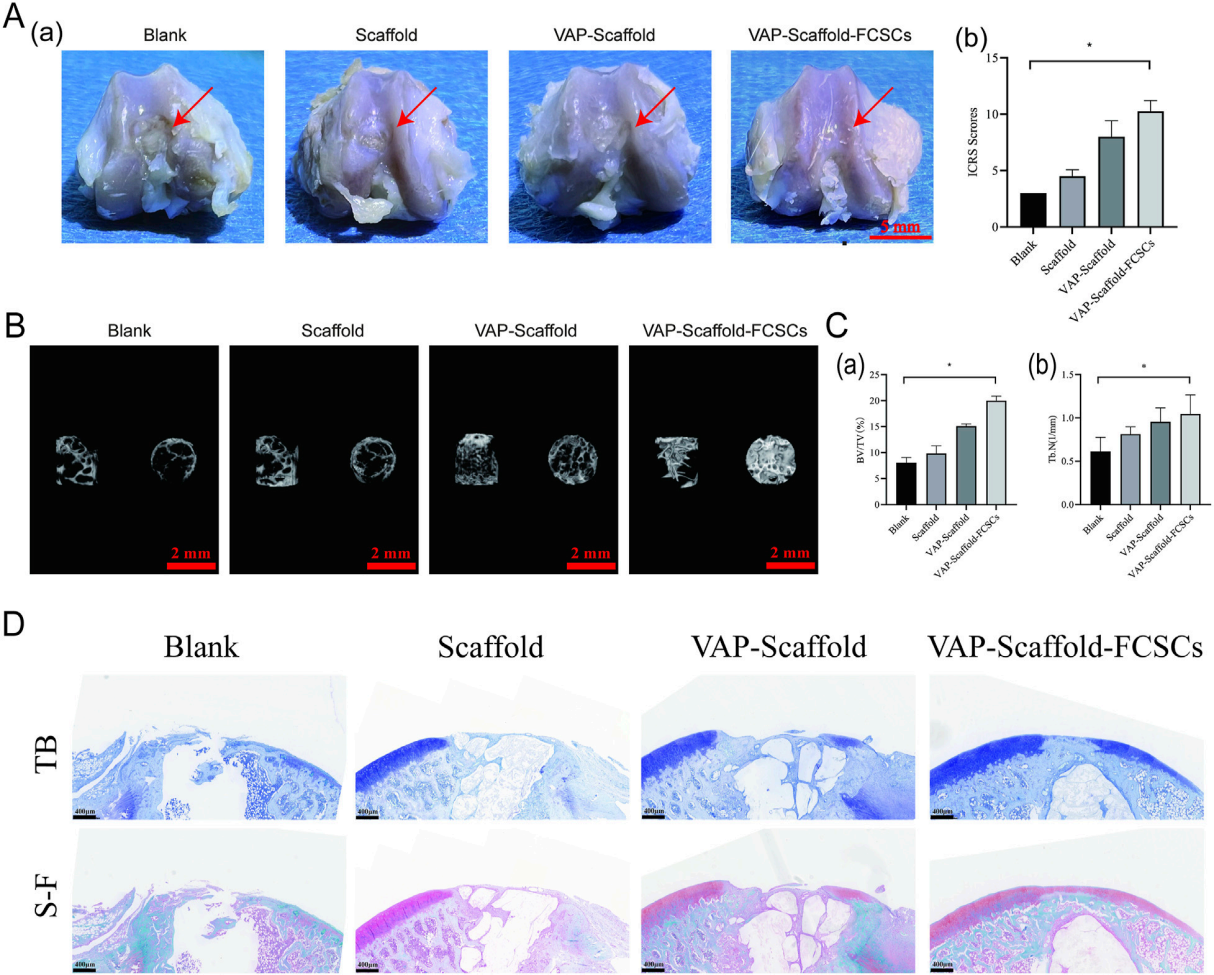
Evaluation of Osteochondral Defect Regeneration. **(A)** Macro-scopic observation of repaired articular defect regions and ICRS scoring analysis. Left: Representative images of repair outcomes, with VAP-Scaffold-FCSCs group showing best effect (scale bar = 5 mm). Right: ICRS scoring results, with significantly higher scores in VAP-Scaffold-FCSCs group compared to Blank group (*p < 0.05). **(B)** Three-dimensional micro-CT reconstructions of defect sites, showing lateral and top views of cylindrical defect regions for each group. **(C)** Quantitative analysis of micro-CT data. Left: Bone volume fraction (BV/TV). Right: Trabecular number (Tb.N), with VAP-Scaffold-FCSCs group showing significantly higher values for both parameters compared to Blank group (*p < 0.05). **(D)** Histological staining of osteochondral defects at 8 weeks post-implantation. TB: Toluidine blue staining for glycosaminoglycan (blue). S–F: Safranin O/Fast Green staining for glycosaminoglycans (red/orange-red) and collagen/osteocytes (green) (Scale bar = 400 µm). * and ** represent *p* < 0.01 and *p* < 0.001 respectively.

H&E staining of the heart, liver, spleen, lungs, and kidneys is a widely accepted method for evaluating systemic toxicity (Yamashita et al., 2023; Zuo et al., 2022). Our analysis revealed no significant pathological changes across all groups, suggesting that the scaffolds did not induce systemic toxicity (Supplementary Figure S1). In this study, SD rats were employed as an osteochondral defect model, with created defects of 2 mm in diameter and depth, exceeding the natural repair capabilities of articular tissue (Meng et al., 2020; Wang et al., 2022b). We implanted triphasic PLA/PCL-PLGA/Mg(OH)_2_ scaffolds modified with polydopamine, VAP, or VAP loaded with FCSCs into the defect sites. After 8 weeks, gross observation and ICRS scoring revealed that the VAP-Scaffold-FCSCs group achieved the best repair outcome (Figure 7A). Three-dimensional micro-CT reconstructions corroborated these findings, By the eighth week post-surgery, all groups exhibited varying degrees of bone repair. The control group showed significantly poorer bone repair compared to the experimental groups. Good bone repair was observed in all experimental groups, with the VAP-Scaffold-FCSCs group displaying notably superior bone remodeling. Statistical analysis also indicated that this group had the largest proportion of bone repair area and the highest number of trabeculae. (Figure 7B). Statistical analysis demonstrated significantly higher percentages of newly formed bone volume and trabecular numbers in the VAP-Scaffold-FCSCs group compared to the blank group (Figure 7C).

Histological evaluation further supported these results, toluidine blue staining showed the formation of new cartilage and fibrous tissue in all groups. The Blank group primarily exhibited new fibrous tissue with fissures. The Scaffold group showed extensive fibrous tissue covering the defect. The VAP-Scaffold group had a small amount of new cartilage and a large amount of fibrous tissue. The VAP-Scaffold-FCSCs group demonstrated the best outcome, with the thickest and most intensely stained cartilage layer. Safranin O-Fast Green staining further confirmed the findings: the Blank group showed almost no red proteoglycan, while the Scaffold and VAP-Scaffold groups displayed a small amount of red proteoglycan. The VAP-Scaffold-FCSCs group exhibited a complete layer of red proteoglycan with the strongest and thickest staining, and new green bone tissue was visible beneath the cartilage. These results indicate that the VAP-Scaffold-FCSCs enhance cartilage and subchondral bone regeneration. (Figure 7D). Satisfactory improvement was made as a result of the optimized pore size in the cartilage layer of the scaffold, thus providing a proper microenvironment for FCSCs. The addition of an isolation layer, behaving like the calcified part, prevented vascular and bone growth inside the cartilage area, ensuring the desired hypoxic conditions important for maintaining the chondrocyte phenotype and matrix deposition (Da et al., 2013). The administration of the VAP-Scaffold-FCSCs group resulted in even superior bone formation, which may be related to the larger pore size in the osseous layer, which promoted osteogenic differentiation. The collaboration of VAP’s biological functions in bone and cartilage systems with the scaffold structures in a tissue repair system was notable. The porous architecture designed into both the cartilage and bone layers, combined with the synergism of FCSCs with VAP, provided favorable conditions for chondral regeneration and repair.

## 4 Conclusion

In this study, we developed a novel tri-phasic PLA/PCL-PLGA/Mg(OH)_2_-VAP scaffold with an osteochondral barrier layer for osteochondral defect treatment. The scaffold features optimized pore sizes for cartilage and bone layers, and incorporates VAP, a bioactive peptide, to enhance regenerative potential. Cytocompatibility tests demonstrated the scaffold’s biocompatibility and its ability to induce chondrogenic and osteogenic differentiation of FCSCs. Preclinical studies validated the efficacy of the FCSC-loaded scaffold in repairing osteochondral defects.

Our results consistently show improved osteogenic and chondrogenic repair both *in vitro* and *in vivo*, attributable to the hierarchical structure of the composite scaffold. The osteochondral barrier layer proved crucial in creating distinct microenvironments for cartilage and bone tissue regeneration. The incorporation of VAP synergistically enhanced the scaffold’s capacity to support osteochondral unit regeneration.

This novel scaffold design, combined with FCSC integration, represents a promising approach for osteochondral defect treatment. Our study provides a foundation for developing tissue-engineered osteochondral constructs and paves the way for future translational research aimed at clinical applications.

## Data Availability

The raw data supporting the conclusions of this article will be made available by the authors, without undue reservation.
