# Supplementary material for: Application of polydopamine-modified triphasic PLA/PCL-PLGA/Mg(OH)2-velvet antler polypeptides scaffold loaded with fibrocartilage stem cells for the repair of osteochondral defects
Source: Front Bioeng Biotechnol. 2024 Sep 19;12:1460623. doi: 10.3389/fbioe.2024.1460623 (PMC11450761; doi:10.3389/fbioe.2024.1460623)
Supplement: Supplementary file 1 [file DataSheet1.DOCX]

**Supplementary Table 1** Compressive Modulus of the scaffolds

| Scaffold | Compressive Modulus |
| --- | --- |
| PLA/PCL-VAP | 65.5044 ± 1.98451 MPa |
| PLGA/Mg(OH)_2_-VAP | 20.7086 ± 0.63024 MPa |

**
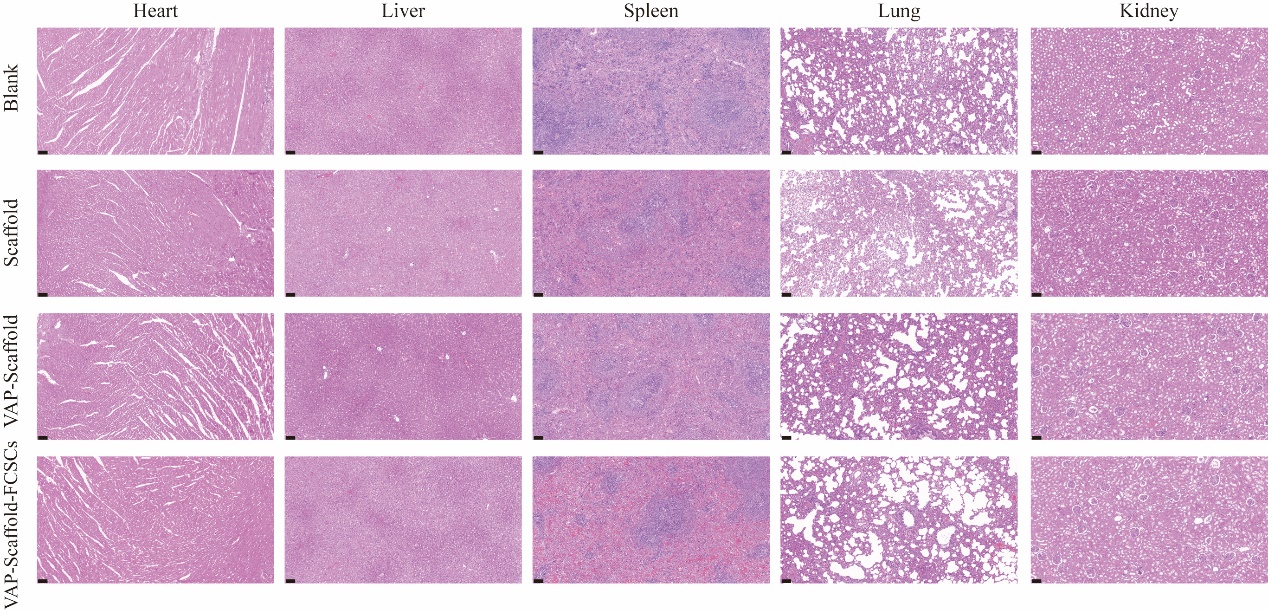
**

**Supplementary Figure 1**. H&E staining of the heart, liver, spleen, lung, and kidney. No significant inflammatory cell infiltration or pathological changes were observed in the organs of all groups (Scale bar = 100 µm).
